# 4-(2,4-Dichlorophenyl)-2-(1*H*-indol-3-yl)-6-(2-pyridyl)-1,4-dihydropyridine-4-carbonitrile

**DOI:** 10.1107/S1600536808027669

**Published:** 2008-09-06

**Authors:** P. Ramesh, A. Subbiahpandi, P. Thirumurugan, Paramasivan T. Perumal, M. N. Ponnuswamy

**Affiliations:** a Department of Physics, Presidency College (Autonomous), Chennai 600 005, India; b Organic Chemistry Division, Central Leather Research Institute, Adyar, Chennai 600 020, India; c Centre of Advanced Study in Crystallography and Biophysics, University of Madras, Guindy Campus, Chennai 600 025, India

## Abstract

The title compound, C_25_H_16_Cl_2_N_4_, has intra­molecular N—H⋯N and C—H⋯Cl hydrogen bonds. In the crystal structure, mol­ecules are linked through N—H⋯N hydrogen bonds, forming a centrosymmetric *R*_2_^2^(16) dimer.

## Related literature

For related literature, see: Beddoes *et al.* (1986 ▶); Bernstein *et al.* (1995 ▶); Harris & Uhle (1960 ▶); Ho *et al.* (1986 ▶); Rajeswaran *et al.* (1999 ▶); Stevenson *et al.* (2000 ▶).
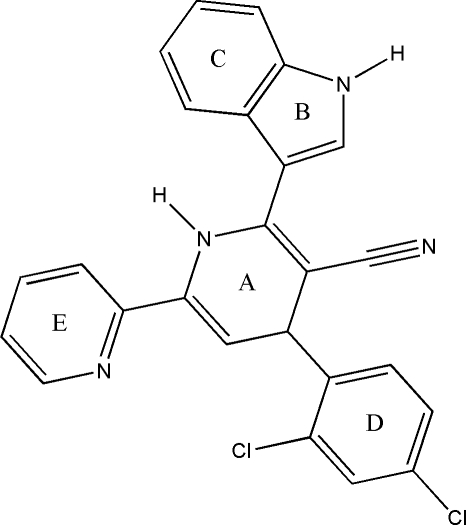


## Experimental

### 

#### Crystal data

C_25_H_16_Cl_2_N_4_
*M* *_r_* = 443.32
Triclinic, 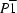

*a* = 8.0158 (9) Å
*b* = 10.0261 (12) Å
*c* = 14.3653 (17) Å
α = 72.260 (6)°
β = 79.420 (6)°
γ = 78.224 (6)°
*V* = 1067.3 (2) Å^3^
*Z* = 2
Mo *K*α radiation
μ = 0.33 mm^−1^
*T* = 298 (2) K
0.35 × 0.32 × 0.28 mm

#### Data collection

Bruker APEXII CCD area-detector diffractometer
Absorption correction: multi-scan (*SADABS*; Sheldrick, 2001 ▶) *T*_min_ = 0.895, *T*_max_ = 0.915
12383 measured reflections
3707 independent reflections
3172 reflections with *I* > 2σ(*I*)
*R*_int_ = 0.023

#### Refinement

*R*[*F* ^2^ > 2σ(*F* ^2^)] = 0.044
*wR*(*F* ^2^) = 0.140
*S* = 1.08
3707 reflections
288 parameters
H atoms treated by a mixture of independent and constrained refinement
Δρ_max_ = 0.61 e Å^−3^
Δρ_min_ = −0.64 e Å^−3^

### 

Data collection: *APEX2* (Bruker, 2004 ▶); cell refinement: *APEX2*; data reduction: *SAINT* (Bruker, 2004 ▶); program(s) used to solve structure: *SHELXS97* (Sheldrick, 2008 ▶); program(s) used to refine structure: *SHELXL97* (Sheldrick, 2008 ▶); molecular graphics: *ORTEP-3* (Farrugia, 1997 ▶); software used to prepare material for publication: *SHELXL97* and *PLATON* (Spek, 2003 ▶).

## Supplementary Material

Crystal structure: contains datablocks global, I. DOI: 10.1107/S1600536808027669/bt2767sup1.cif

Structure factors: contains datablocks I. DOI: 10.1107/S1600536808027669/bt2767Isup2.hkl

Additional supplementary materials:  crystallographic information; 3D view; checkCIF report

## Figures and Tables

**Table 1:** Hydrogen-bond geometry (Å, °)

*D*—H⋯*A*	*D*—H	H⋯*A*	*D*⋯*A*	*D*—H⋯*A*
N1—H1⋯N29	0.85 (3)	2.21 (3)	2.638 (2)	111 (2)
C4—H4⋯Cl1	0.98	2.56	3.114 (2)	116
N14—H14⋯N17^i^	0.84 (3)	2.14 (3)	2.937 (3)	158 (2)

Symmetry code: (i) 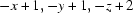.

**Fig. 1.**
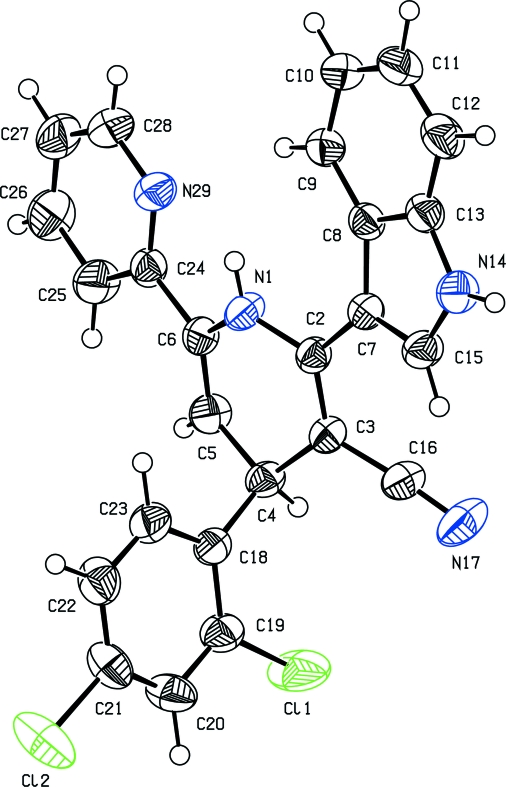
Perspective view of the molecules showing the thermal ellipsoids are drawn at 50% probability level. The H atoms are shown as small circles of arbitrary radii.

## supplementary crystallographic information

### Comment

Indole derivatives are used as bioactive drugs (Stevenson *et al.*, 2000) and they exibit anti-allergic, central nervous system depressant and muscle relaxant properties (Harris & Uhle 1960; Ho *et al.*, 1986). Indoles also have been proved to display high aldose reductase inhibitory activity (Rajeswaran *et al.*., 1999). In view of these biological importance, an X-ray diffraction study of the title compound, (I), was carrid out.

The pyridine ring A adopts a planar conformation. The planar indole ring system and the pyridine ring E lie in the plane of pyridine ring A. The bond angle of (C3—C16—N17) 174.7 (2)° shows linear character of the cyano group, a feature observed in carbonitrile compounds. The sum of the angles at N1 of the pyridine ring (358.16°) is in accordance with *sp*^2^ hybridization (Beddoes *et al.*, 1986).

The crystal structure is stabilized by N—H···N interactions. Atom N1 donates a proton to atom N29 and it forms a S(5) ring motif (Bernstein *et al.*, 1995). The molecules at positions (*x*, *y*, *z*) and (1 - *x*,1 - *y*,-*z*) form a cyclic centrosymmetric *R*_2_^2^(16) dimer through N14—H14···N17 hydrogen bonds.

### Experimental

A mixture of 3-cyanoacetyl indole (1 mmol), 2,4 dichlorobenzaldehyde (1 mmol) and 2-acetyl pyridine (1 mmol) in 5 g m of ammounim acetate under neat condition was refluxed for 6–8 h. After the completion of the reaction (as monitored by TLC), it was poured into water and extracted with ethyl acetate. The organic layer was dried over sodium sulfate and concentrated under vacuo. The crude product was chromatographed and isolated in 80% yield (90:10, petroleum ether: ethyl acetate). The crude was recrystallized in ethanol

### Refinement

H atoms bonded to C were positioned geometrically (C—H = 0.93–0.98 Å) and allowed to ride on their parent atoms, with *U*_iso_(H) = 1.2*U*_eq_(C). The H atoms bonded to N were freely refined.

### Crystal data

**Table:**

C_25_H_16_Cl_2_N_4_	*Z* = 2
*M**_r_* = 443.32	*F*(000) = 456
Triclinic, *P*1	*D*_x_ = 1.379 Mg m^−^^3^
Hall symbol: -P 1	Mo *K*α radiation, λ = 0.71073 Å
*a* = 8.0158 (9) Å	Cell parameters from 2900 reflections
*b* = 10.0261 (12) Å	θ = 2.2–25.0°
*c* = 14.3653 (17) Å	µ = 0.33 mm^−^^1^
α = 72.260 (6)°	*T* = 298 K
β = 79.420 (6)°	Block, yellow
γ = 78.224 (6)°	0.35 × 0.32 × 0.28 mm
*V* = 1067.3 (2) Å^3^	

### Data collection

**Table:**

Bruker APEX2 CCD area-detector diffractometer	3707 independent reflections
Radiation source: fine-focus sealed tube	3172 reflections with *I* > 2σ(*I*)
graphite	*R*_int_ = 0.023
ω and φ scans	θ_max_ = 25.0°, θ_min_ = 2.2°
Absorption correction: multi-scan (SADABS; Sheldrick, 2001)	*h* = −9→7
*T*_min_ = 0.895, *T*_max_ = 0.915	*k* = −11→11
12383 measured reflections	*l* = −17→16

### Refinement

**Table:**

Refinement on *F*^2^	Primary atom site location: structure-invariant direct methods
Least-squares matrix: full	Secondary atom site location: difference Fourier map
*R*[*F*^2^ > 2σ(*F*^2^)] = 0.044	Hydrogen site location: inferred from neighbouring sites
*wR*(*F*^2^) = 0.140	H atoms treated by a mixture of independent and constrained refinement
*S* = 1.08	*w* = 1/[σ^2^(*F*_o_^2^) + (0.0719*P*)^2^ + 0.5551*P*] where *P* = (*F*_o_^2^ + 2*F*_c_^2^)/3
3707 reflections	(Δ/σ)_max_ = 0.001
288 parameters	Δρ_max_ = 0.61 e Å^−^^3^
0 restraints	Δρ_min_ = −0.64 e Å^−^^3^

### Special details

**Table:**

Geometry. All e.s.d.'s (except the e.s.d. in the dihedral angle between two l.s. planes) are estimated using the full covariance matrix. The cell e.s.d.'s are taken into account individually in the estimation of e.s.d.'s in distances, angles and torsion angles; correlations between e.s.d.'s in cell parameters are only used when they are defined by crystal symmetry. An approximate (isotropic) treatment of cell e.s.d.'s is used for estimating e.s.d.'s involving l.s. planes.
Refinement. Refinement of *F*^2^ against ALL reflections. The weighted *R*-factor *wR* and goodness of fit *S* are based on *F*^2^, conventional *R*-factors *R* are based on *F*, with *F* set to zero for negative *F*^2^. The threshold expression of *F*^2^ > σ(*F*^2^) is used only for calculating *R*-factors(gt) *etc*. and is not relevant to the choice of reflections for refinement. *R*-factors based on *F*^2^ are statistically about twice as large as those based on *F*, and *R*- factors based on ALL data will be even larger.

### Fractional atomic coordinates and isotropic or equivalent isotropic displacement parameters (Å^2^)

**Table:**

	*x*	*y*	*z*	*U*_iso_*/*U*_eq_	
Cl1	0.65524 (10)	0.88516 (11)	0.55182 (6)	0.0861 (3)	
Cl2	1.32340 (10)	0.67807 (10)	0.50447 (5)	0.0832 (3)	
N1	0.7936 (2)	0.98325 (19)	0.91511 (14)	0.0411 (4)	
H1	0.821 (3)	0.993 (3)	0.966 (2)	0.055 (8)*	
C2	0.7078 (3)	0.8757 (2)	0.92099 (15)	0.0342 (4)	
C3	0.6640 (3)	0.8668 (2)	0.83578 (15)	0.0362 (5)	
C4	0.7222 (3)	0.9620 (2)	0.73464 (15)	0.0373 (5)	
H4	0.6248	0.9910	0.6966	0.045*	
C5	0.7668 (3)	1.0937 (2)	0.74732 (16)	0.0412 (5)	
H5	0.7692	1.1738	0.6936	0.049*	
C6	0.8027 (3)	1.0996 (2)	0.83236 (15)	0.0359 (5)	
C7	0.6707 (3)	0.7807 (2)	1.01918 (15)	0.0357 (5)	
C8	0.6776 (3)	0.8008 (2)	1.11452 (15)	0.0355 (4)	
C9	0.7107 (3)	0.9087 (2)	1.14899 (17)	0.0426 (5)	
H9	0.7337	0.9946	1.1049	0.051*	
C10	0.7087 (3)	0.8860 (3)	1.24827 (18)	0.0515 (6)	
H10	0.7316	0.9572	1.2709	0.062*	
C11	0.6732 (3)	0.7589 (3)	1.31624 (18)	0.0553 (6)	
H11	0.6743	0.7465	1.3830	0.066*	
C12	0.6369 (3)	0.6519 (3)	1.28621 (17)	0.0523 (6)	
H12	0.6108	0.5677	1.3314	0.063*	
C13	0.6405 (3)	0.6741 (2)	1.18565 (16)	0.0408 (5)	
N14	0.6121 (3)	0.5841 (2)	1.13699 (14)	0.0475 (5)	
H14	0.586 (3)	0.503 (3)	1.1635 (19)	0.050 (7)*	
C15	0.6302 (3)	0.6468 (2)	1.03934 (16)	0.0430 (5)	
H15	0.6172	0.6054	0.9919	0.052*	
C16	0.5542 (3)	0.7736 (2)	0.83477 (16)	0.0433 (5)	
N17	0.4654 (3)	0.7028 (2)	0.82628 (17)	0.0636 (6)	
C18	0.8703 (3)	0.8858 (2)	0.67753 (15)	0.0365 (5)	
C19	0.8534 (3)	0.8496 (3)	0.59441 (17)	0.0455 (5)	
C20	0.9908 (3)	0.7851 (3)	0.54127 (18)	0.0548 (6)	
H20	0.9761	0.7629	0.4853	0.066*	
C21	1.1491 (3)	0.7547 (3)	0.57292 (17)	0.0522 (6)	
C22	1.1726 (3)	0.7853 (3)	0.65559 (18)	0.0537 (6)	
H22	1.2802	0.7628	0.6770	0.064*	
C23	1.0333 (3)	0.8503 (3)	0.70659 (17)	0.0474 (6)	
H23	1.0492	0.8713	0.7628	0.057*	
C24	0.8451 (3)	1.2263 (2)	0.85058 (16)	0.0382 (5)	
C25	0.8700 (4)	1.3490 (2)	0.77569 (19)	0.0540 (6)	
H25	0.8621	1.3543	0.7108	0.065*	
C26	0.9063 (4)	1.4627 (3)	0.7986 (2)	0.0614 (7)	
H26	0.9234	1.5458	0.7495	0.074*	
C27	0.9171 (3)	1.4520 (3)	0.8944 (2)	0.0580 (7)	
H27	0.9407	1.5276	0.9119	0.070*	
C28	0.8923 (3)	1.3271 (3)	0.9640 (2)	0.0560 (6)	
H28	0.9000	1.3201	1.0292	0.067*	
N29	0.8574 (3)	1.2145 (2)	0.94397 (14)	0.0465 (5)	

### Atomic displacement parameters (Å^2^)

**Table:**

	*U*^11^	*U*^22^	*U*^33^	*U*^12^	*U*^13^	*U*^23^
Cl1	0.0591 (4)	0.1440 (8)	0.0794 (5)	0.0098 (4)	−0.0343 (4)	−0.0684 (6)
Cl2	0.0690 (5)	0.1084 (7)	0.0464 (4)	0.0200 (4)	0.0061 (3)	−0.0138 (4)
N1	0.0546 (11)	0.0394 (10)	0.0346 (10)	−0.0202 (8)	−0.0125 (8)	−0.0056 (8)
C2	0.0346 (10)	0.0320 (10)	0.0376 (11)	−0.0075 (8)	−0.0042 (8)	−0.0106 (8)
C3	0.0383 (11)	0.0334 (11)	0.0389 (11)	−0.0084 (8)	−0.0066 (9)	−0.0105 (9)
C4	0.0413 (11)	0.0390 (11)	0.0337 (11)	−0.0072 (9)	−0.0105 (8)	−0.0094 (9)
C5	0.0524 (13)	0.0329 (11)	0.0370 (12)	−0.0100 (9)	−0.0069 (9)	−0.0051 (9)
C6	0.0386 (11)	0.0312 (10)	0.0374 (11)	−0.0092 (8)	−0.0032 (8)	−0.0074 (9)
C7	0.0372 (11)	0.0341 (10)	0.0370 (11)	−0.0086 (8)	−0.0029 (8)	−0.0109 (9)
C8	0.0335 (10)	0.0361 (11)	0.0363 (11)	−0.0051 (8)	−0.0006 (8)	−0.0115 (9)
C9	0.0443 (12)	0.0427 (12)	0.0435 (12)	−0.0110 (10)	0.0017 (9)	−0.0175 (10)
C10	0.0494 (13)	0.0661 (16)	0.0489 (14)	−0.0139 (12)	0.0008 (10)	−0.0314 (12)
C11	0.0545 (14)	0.0778 (18)	0.0368 (13)	−0.0136 (13)	0.0003 (10)	−0.0224 (12)
C12	0.0555 (14)	0.0582 (15)	0.0368 (12)	−0.0130 (12)	0.0014 (10)	−0.0054 (11)
C13	0.0429 (12)	0.0410 (12)	0.0377 (12)	−0.0095 (9)	0.0002 (9)	−0.0109 (9)
N14	0.0648 (13)	0.0363 (10)	0.0413 (11)	−0.0213 (9)	−0.0015 (9)	−0.0049 (8)
C15	0.0532 (13)	0.0388 (12)	0.0398 (12)	−0.0147 (10)	−0.0043 (10)	−0.0115 (9)
C16	0.0558 (13)	0.0381 (12)	0.0395 (12)	−0.0119 (10)	−0.0126 (10)	−0.0092 (9)
N17	0.0899 (17)	0.0533 (13)	0.0592 (14)	−0.0350 (12)	−0.0293 (12)	−0.0053 (10)
C18	0.0427 (11)	0.0357 (11)	0.0320 (11)	−0.0119 (9)	−0.0076 (8)	−0.0054 (8)
C19	0.0463 (12)	0.0546 (14)	0.0384 (12)	−0.0064 (10)	−0.0130 (10)	−0.0138 (10)
C20	0.0632 (16)	0.0678 (16)	0.0349 (12)	−0.0037 (13)	−0.0084 (11)	−0.0195 (11)
C21	0.0511 (14)	0.0557 (15)	0.0375 (13)	−0.0017 (11)	0.0017 (10)	−0.0040 (11)
C22	0.0407 (12)	0.0694 (17)	0.0472 (14)	−0.0072 (11)	−0.0095 (10)	−0.0094 (12)
C23	0.0466 (13)	0.0585 (14)	0.0413 (12)	−0.0136 (11)	−0.0109 (10)	−0.0137 (11)
C24	0.0368 (11)	0.0352 (11)	0.0431 (12)	−0.0078 (9)	−0.0011 (9)	−0.0129 (9)
C25	0.0737 (17)	0.0415 (13)	0.0481 (14)	−0.0196 (12)	−0.0043 (12)	−0.0095 (11)
C26	0.0767 (18)	0.0367 (13)	0.0698 (18)	−0.0200 (12)	−0.0008 (14)	−0.0111 (12)
C27	0.0606 (16)	0.0446 (14)	0.0786 (19)	−0.0162 (12)	−0.0004 (13)	−0.0313 (13)
C28	0.0643 (16)	0.0558 (15)	0.0594 (16)	−0.0184 (12)	−0.0043 (12)	−0.0293 (13)
N29	0.0549 (11)	0.0451 (11)	0.0461 (11)	−0.0165 (9)	−0.0044 (9)	−0.0178 (9)

### Geometric parameters (Å, °)

**Table:**

Cl1—C19	1.737 (2)	C12—H12	0.9300
Cl2—C21	1.740 (2)	C13—N14	1.372 (3)
N1—C2	1.367 (3)	N14—C15	1.345 (3)
N1—C6	1.392 (3)	N14—H14	0.84 (3)
N1—H1	0.84 (3)	C15—H15	0.9300
C2—C3	1.366 (3)	C16—N17	1.147 (3)
C2—C7	1.459 (3)	C18—C19	1.385 (3)
C3—C16	1.413 (3)	C18—C23	1.388 (3)
C3—C4	1.526 (3)	C19—C20	1.384 (3)
C4—C5	1.506 (3)	C20—C21	1.371 (4)
C4—C18	1.524 (3)	C20—H20	0.9300
C4—H4	0.9800	C21—C22	1.367 (4)
C5—C6	1.326 (3)	C22—C23	1.379 (3)
C5—H5	0.9300	C22—H22	0.9300
C6—C24	1.487 (3)	C23—H23	0.9300
C7—C15	1.377 (3)	C24—N29	1.331 (3)
C7—C8	1.456 (3)	C24—C25	1.388 (3)
C8—C9	1.407 (3)	C25—C26	1.376 (3)
C8—C13	1.413 (3)	C25—H25	0.9300
C9—C10	1.372 (3)	C26—C27	1.364 (4)
C9—H9	0.9300	C26—H26	0.9300
C10—C11	1.395 (4)	C27—C28	1.369 (4)
C10—H10	0.9300	C27—H27	0.9300
C11—C12	1.370 (4)	C28—N29	1.339 (3)
C11—H11	0.9300	C28—H28	0.9300
C12—C13	1.389 (3)		
			
C2—N1—C6	122.28 (18)	C15—N14—C13	109.48 (19)
C2—N1—H1	120.3 (19)	C15—N14—H14	124.8 (17)
C6—N1—H1	115.6 (19)	C13—N14—H14	125.8 (17)
C3—C2—N1	117.87 (18)	N14—C15—C7	110.8 (2)
C3—C2—C7	125.98 (18)	N14—C15—H15	124.6
N1—C2—C7	116.15 (18)	C7—C15—H15	124.6
C2—C3—C16	122.62 (19)	N17—C16—C3	174.7 (2)
C2—C3—C4	122.64 (17)	C19—C18—C23	116.2 (2)
C16—C3—C4	114.67 (18)	C19—C18—C4	123.25 (19)
C5—C4—C18	111.94 (17)	C23—C18—C4	120.57 (19)
C5—C4—C3	108.98 (16)	C20—C19—C18	122.5 (2)
C18—C4—C3	112.45 (17)	C20—C19—Cl1	116.87 (17)
C5—C4—H4	107.8	C18—C19—Cl1	120.65 (18)
C18—C4—H4	107.8	C21—C20—C19	118.6 (2)
C3—C4—H4	107.8	C21—C20—H20	120.7
C6—C5—C4	122.38 (19)	C19—C20—H20	120.7
C6—C5—H5	118.8	C22—C21—C20	121.4 (2)
C4—C5—H5	118.8	C22—C21—Cl2	119.8 (2)
C5—C6—N1	120.15 (18)	C20—C21—Cl2	118.7 (2)
C5—C6—C24	125.28 (19)	C21—C22—C23	118.6 (2)
N1—C6—C24	114.49 (18)	C21—C22—H22	120.7
C15—C7—C8	105.65 (18)	C23—C22—H22	120.7
C15—C7—C2	125.51 (19)	C22—C23—C18	122.7 (2)
C8—C7—C2	128.75 (18)	C22—C23—H23	118.6
C9—C8—C13	117.36 (19)	C18—C23—H23	118.6
C9—C8—C7	136.58 (19)	N29—C24—C25	122.0 (2)
C13—C8—C7	106.06 (18)	N29—C24—C6	115.44 (18)
C10—C9—C8	119.2 (2)	C25—C24—C6	122.6 (2)
C10—C9—H9	120.4	C26—C25—C24	119.1 (2)
C8—C9—H9	120.4	C26—C25—H25	120.4
C9—C10—C11	121.7 (2)	C24—C25—H25	120.4
C9—C10—H10	119.1	C27—C26—C25	119.2 (2)
C11—C10—H10	119.1	C27—C26—H26	120.4
C12—C11—C10	121.0 (2)	C25—C26—H26	120.4
C12—C11—H11	119.5	C26—C27—C28	118.2 (2)
C10—C11—H11	119.5	C26—C27—H27	120.9
C11—C12—C13	117.3 (2)	C28—C27—H27	120.9
C11—C12—H12	121.3	N29—C28—C27	123.9 (2)
C13—C12—H12	121.3	N29—C28—H28	118.0
N14—C13—C12	128.7 (2)	C27—C28—H28	118.0
N14—C13—C8	107.98 (19)	C24—N29—C28	117.5 (2)
C12—C13—C8	123.3 (2)		
			
C6—N1—C2—C3	15.0 (3)	C8—C13—N14—C15	0.3 (3)
C6—N1—C2—C7	−164.76 (19)	C13—N14—C15—C7	−0.2 (3)
N1—C2—C3—C16	−170.6 (2)	C8—C7—C15—N14	0.0 (3)
C7—C2—C3—C16	9.1 (3)	C2—C7—C15—N14	176.8 (2)
N1—C2—C3—C4	6.2 (3)	C2—C3—C16—N17	173 (3)
C7—C2—C3—C4	−174.09 (19)	C4—C3—C16—N17	−4(3)
C2—C3—C4—C5	−22.1 (3)	C5—C4—C18—C19	−126.6 (2)
C16—C3—C4—C5	154.91 (19)	C3—C4—C18—C19	110.4 (2)
C2—C3—C4—C18	102.6 (2)	C5—C4—C18—C23	52.5 (3)
C16—C3—C4—C18	−80.4 (2)	C3—C4—C18—C23	−70.6 (2)
C18—C4—C5—C6	−104.9 (2)	C23—C18—C19—C20	−1.6 (3)
C3—C4—C5—C6	20.1 (3)	C4—C18—C19—C20	177.5 (2)
C4—C5—C6—N1	−2.5 (3)	C23—C18—C19—Cl1	178.79 (17)
C4—C5—C6—C24	−179.03 (19)	C4—C18—C19—Cl1	−2.1 (3)
C2—N1—C6—C5	−17.2 (3)	C18—C19—C20—C21	0.7 (4)
C2—N1—C6—C24	159.66 (19)	Cl1—C19—C20—C21	−179.7 (2)
C3—C2—C7—C15	19.2 (3)	C19—C20—C21—C22	0.7 (4)
N1—C2—C7—C15	−161.1 (2)	C19—C20—C21—Cl2	−178.27 (19)
C3—C2—C7—C8	−164.7 (2)	C20—C21—C22—C23	−1.1 (4)
N1—C2—C7—C8	15.0 (3)	Cl2—C21—C22—C23	177.89 (19)
C15—C7—C8—C9	180.0 (2)	C21—C22—C23—C18	0.1 (4)
C2—C7—C8—C9	3.3 (4)	C19—C18—C23—C22	1.2 (3)
C15—C7—C8—C13	0.2 (2)	C4—C18—C23—C22	−177.9 (2)
C2—C7—C8—C13	−176.5 (2)	C5—C6—C24—N29	172.5 (2)
C13—C8—C9—C10	1.2 (3)	N1—C6—C24—N29	−4.2 (3)
C7—C8—C9—C10	−178.6 (2)	C5—C6—C24—C25	−7.3 (4)
C8—C9—C10—C11	−0.6 (4)	N1—C6—C24—C25	176.0 (2)
C9—C10—C11—C12	−0.8 (4)	N29—C24—C25—C26	−0.8 (4)
C10—C11—C12—C13	1.4 (4)	C6—C24—C25—C26	179.0 (2)
C11—C12—C13—N14	178.8 (2)	C24—C25—C26—C27	0.0 (4)
C11—C12—C13—C8	−0.7 (4)	C25—C26—C27—C28	0.5 (4)
C9—C8—C13—N14	179.84 (19)	C26—C27—C28—N29	−0.2 (4)
C7—C8—C13—N14	−0.3 (2)	C25—C24—N29—C28	1.1 (3)
C9—C8—C13—C12	−0.6 (3)	C6—C24—N29—C28	−178.8 (2)
C7—C8—C13—C12	179.3 (2)	C27—C28—N29—C24	−0.6 (4)
C12—C13—N14—C15	−179.2 (2)		

### Hydrogen-bond geometry (Å, °)

**Table:**

*D*—H···*A*	*D*—H	H···*A*	*D*···*A*	*D*—H···*A*
N1—H1···N29	0.85 (3)	2.21 (3)	2.638 (2)	111 (2)
C4—H4···Cl1	0.98	2.56	3.114 (2)	116.
N14—H14···N17^i^	0.84 (3)	2.14 (3)	2.937 (3)	158 (2)

Symmetry codes:  (i) −*x*+1, −*y*+1, −*z*+2.
